# Nostrum Certifiers

**Published:** 1846-10

**Authors:** 


*Nostrum certifiers.*—Under this head the editor of the Western Lancet is collecting, from various quarters, the names of medical men who have given certificates of the value of secret compounds. Physicians have been too careless on this point. Sometimes their good nature has led them to yield to the importunities of some acquaintance who wishes to speculate on the credulity of the public. Oftener they are deceived by false assurances that their certificate is to be used only to a certain extent, or with certain qualifications. In either case there can be no excuse. The principle is radically wrong, and although the results concern the public, who are to be duped, vastly more than the physician, yet, he is aware of the deception, and is responsible, not only for any influence which he may exert in furthering its success, but for withholding all judicious counteracting influences. While upon this subject we cannot but remark, that considering the pecuniary enticements furnished by the various methods of quackery, it is highly creditable to the profession that so few desert the path of integrity for these shorter and easier roads to wealth. The number of those who have been deluded into certifying to nostrums is by no means large, but the number of those who have deliberately and openly adopted the practices of quackery, is quite insignificant. We think this fact may be cited as affording striking evidence of the honorable character which belongs to the medical profession as a class.

